# A Randomized Controlled Trial of Two Different Macronutrient Profiles on Weight, Body Composition and Metabolic Parameters in Obese Adolescents Seeking Weight Loss

**DOI:** 10.1371/journal.pone.0151787

**Published:** 2016-03-29

**Authors:** Helen Truby, Kimberley Baxter, Robert S. Ware, Diane E. Jensen, John W. Cardinal, Janet M. Warren, Lynne Daniels, Peter S. W. Davies, Paula Barrett, Michelle L. Blumfield, Jennifer A. Batch

**Affiliations:** 1 Department of Nutrition and Dietetics, Monash University, Clayton, Victoria, Australia; 2 Children’s Nutrition Research Centre, The University of Queensland, Herston, Queensland, Australia; 3 UQ Child Health Research Centre, School of Medicine, The University of Queensland, South Brisbane, Queensland, Australia; 4 School of Public Health, The University of Queensland, Herston, Queensland, Australia; 5 Children’s Health Queensland Hospital and Health Service, Department of Endocrinology and Diabetes, Lady Cilentro Children’s Hospital, South Brisbane, Queensland, Australia; 6 Chemical Pathology, Pathology Queensland, Herston, Queensland, Australia; 7 School of Exercise and Nutrition Sciences, Queensland University of Technology, Kelvin Grove, Queensland, Australia; 8 Pathways Health and Research Centre, West End, Brisbane, Queensland, Australia; Texas A&M University, UNITED STATES

## Abstract

**Objective:**

Adolescent obesity is difficult to treat and the optimal dietary pattern, particularly in relation to macronutrient composition, remains controversial. This study tested the effect of two structured diets with differing macronutrient composition versus control, on weight, body composition and metabolic parameters in obese adolescents.

**Design:**

A randomized controlled trial conducted in a children’s hospital.

**Methods:**

Eighty seven obese youth (means: age 13.6 years, BMI z-score 2.2, waist: height ratio 0.65, 69% female) completed a psychological preparedness program and were then randomized to a short term ‘structured modified carbohydrate’ (SMC, 35% carbohydrate; 30% protein; 35% fat, n = 37) or a ‘structured low fat’ (SLF, 55% carbohydrate; 20% protein; 25% fat, n = 36) or a wait listed control group (n = 14). Anthropometric, body composition and biochemical parameters were measured at randomization and after 12 weeks, and analyzed under the intention to treat principle using analysis of variance models.

**Results:**

After 12 weeks, data was collected from 79 (91%) participants. BMI z-scores were significantly lower in both intervention groups compared to control after adjusting for baseline values, SLF vs. control, mean difference = -0.13 (95%CI = -0.18, -0.07), P<0.001; SMC vs. control, -0.14 (-0.19, -0.09), P<0.001, but there was no difference between the two intervention diet groups: SLF vs. SMC, 0.00 (-0.05, 0.04), P = 0.83.

**Conclusions:**

Both dietary patterns resulted in similar changes in weight, body composition and metabolic improvements compared to control. The use of a structured eating system which allows flexibility but limited choices can assist in weight change and the rigid application of a low fat eating pattern is not exclusive in its efficacy.

**Trial Registration:**

International Clinical Trials Registry ISRCTN49438757

## Introduction

Obesity in children and adolescents is of particular concern given the high prevalence worldwide and the concomitant increased risk of developing comorbidities including cardiovascular diseases (CVD) and diabetes [1]. It has been previously shown that the risk of a CVD event in adulthood was significantly higher for every 1 unit increase in body mass index (BMI) z-score for 7–13 year old children [2]. Therefore, it would seem that modest decreases in childhood BMI could be an effective secondary prevention strategy for CVD [3]. At the most basic level, reductions in BMI can be achieved by being in sustained negative energy balance (i.e. reduction in energy intake and/or increased energy expenditure). However recent Cochrane reviews [4, 5] for the treatment of paediatric obesity demonstrate the relative lack of evidence for effective treatment, particularly in children 12 years and older and emphasised the need for research that assesses the effectiveness of dietary interventions on responsive outcomes such as weight and lipid profile.

A dietary pattern low in fat (25–30% energy) is recommended internationally as a way to minimise CVD risk, and is aligned with dietary guidelines that enable choice across a wide variety of foods. A systematic review across 30 countries robustly demonstrates that dietary fat intake in children and adolescents are not aligned with recommendations [6]. Population health surveys in the US and UK, that include children >2 years of age report dietary fat intake around 34–36% of total energy intake [7, 8]. In Australian national surveillance data collected in 2011–12 using a single 24 hour recall, reported dietary fat in 9–13 and 14–18 years olds was 31.4% and 31.8%, respectively [9]. In order to further guide the population, Australia provides a proportional range of fat intake for children >2 years of age, the lower level of 20% and upper acceptable level of 35% of energy intake [10]. Thus reducing consumption of dietary fat to align with dietary guidelines is an obvious target for a weight and cardiovascular risk reduction strategy.

Carbohydrate intake has been a target for weight reduction programs as it is the largest proportional contributor to overall energy intake (48–54%) in children and adolescents [8, 9]. Strategies that have been tried in adults include very low carbohydrate diets which have proved to be efficacious, despite initial scepticism that a higher fat content would adversely affect lipid profile [11]. A recent meta-analysis comparing low fat versus very low carbohydrate diets in adults concluded that low carbohydrate approaches may be equally, if not more effective, in reducing weight and cardiovascular risk parameters after twelve months [12]. To date, most of the low carbohydrate approaches tested have at least initial periods of severe restriction (20-50g/day) resulting in ketosis. A study in 12–18 year olds reported similar findings, with a very low carbohydrate (20g/day), high protein (2–2.5g protein/kg ideal body weight/day) diet compared to a low fat (30% energy) diet being superior in terms of weight loss, safety and efficacy after 13 weeks [13]. In younger children (7–12 years), modification of dietary carbohydrate was as effective as ‘standard care’ (50–55% CHO, 10–15% protein, 30% fat) in terms of weight loss [14]. However, following a very low carbohydrate unrestricted protein diet which induced ketosis was difficult for the children to adhere to, compared to a low glycaemic index carbohydrate dietary pattern and therefore not a sustainable strategy to be promoted at a public health level [14].

Dietary protein needs to be taken into consideration and obviously alters proportionally in line with changes in other macronutrients. Ervin and Ogden [8] reported a rise in dietary protein in the US (boys to 14.7% and 14.3% for girls) in 2010, which was slightly less than Australian children who consume between 16–17% protein [9]. Gosby’s et al. (2014) systematic review demonstrated that a relationship exists between higher protein intakes with lower energy intakes in individuals aged 17–80 years, suggestive that if we derive more protein in our diets it will help weight control longer term [15]. There is also some evidence across the lifespan that loss of lean tissue could be minimized by a higher protein intake during a weight loss attempt [16].

The ‘low fat’ regimen was based on the proportional macronutrient composition between the lower and upper acceptable ranges of macronutrients as defined by the Australian guidelines [10]. We hypothesized that a modest reduction in carbohydrate combined with a greater protein intake could provide an optimal combination of macronutrients to our target group and that either dietary pattern had potential to be adopted at a public health level. This approach was tested in a ‘proof of concept’ phase that demonstrated the acceptability of using a very structured food exchange menu approach alongside a lowering of carbohydrate to 35% energy [17]. This test phase additionally highlighted that the adolescents presenting at a tertiary hospital were unwilling or unable to engage in physical activity of sufficient intensity or duration to induce weight loss. Therefore, we aimed to compare the impact on weight of two different macronutrient dietary patterns and the short term effect on a range of biochemical parameters compared to an untreated control group. We test two hypotheses (1) there will be a change in BMI z-score between the intervention diets and control; and (2) there will be a change in mean BMI z-score between the low fat and the reduced carbohydrate group. Recognising the many adverse psychological effects of childhood obesity [18], completion of a formal psychological preparatory group program was a necessary initial step so that all participants were in good psychological health, and similarly prepared for lifestyle change.

## Materials and Methods

This randomized controlled trial took place at a tertiary children’s hospital in Brisbane, Australia. The ‘Eat Smart’ protocol has been published in full (see S1 Text) [19]. The study was conducted according to the guidelines laid down in the Declaration of Helsinki and all procedures involving human subjects were approved by the Royal Children’s Hospital & Health Service District Ethics Committee (05/02/2008; #2008/005) that included the inclusion of a wait listed control. Written informed consent was obtained from all parents/guardians and assent by their child. The ‘Eat Smart’ study is registered with the International Clinical Trials Registry (ISRCTN49438757; http://apps.who.int/trialsearch/). Recruitment commenced three months prior to registration due to administrative delays in payment processing. A CONSORT 2010 checklist for this trial is presented in S1 Table. The authors confirm that all ongoing and related trials for this intervention are registered.

### Eligibility

Participants were recruited after referral from a health professional and were initially screened via telephone. Inclusion criteria: participants aged 10–17 years with a BMI >90^th^ percentile, as defined by Centre for Disease Control growth charts (CDC 2000) [20]. The World Health Organisation defines adolescent development as between 10–19 years. In the tertiary hospital sector in Australia, adolescents are seen until the age of 17 then moved to adult hospital. These factors drove our inclusion age range. Exclusion criteria were use of stimulants or psychotropic drugs known to alter body composition or metabolism including insulin sensitisers (eg. Biguanides such as metformin), glucocorticoids and thyroxin. Those with obesity related to a medical condition (eg. Prader Willi Syndrome) and those with Type 1 diabetes or complex food allergies were excluded. After completing a six week preparatory phase (FRIENDS for life^TM^ program [21]), all participants completed the Anxiety Disorder Interview Schedule (ADIS) with a set of additional short questions on eating disorder behaviours in a one to one interview with a clinical psychologist. In the event that the psychological interview suggested the presence of significant or clinically relevant levels of anxiety, depression, dysthymia, or other mental health co-morbidity, participants were excluded from further participation and referred for clinical psychological services. The ADIS interview was repeated at the end of the dietary intervention phase (after week 12) to ensure that no psychological harm was induced by taking part in the study protocol.

At baseline (week 0), all participants attended the hospital after an overnight fast, they were weighed (kg) and measured (height to the nearest complete mm) and BMI z-score calculated by the LMS method [22]. Blood pressure was measured using an automatic blood pressure device (Vital Signs Monitor—Model No. 53N00, Welch Allyn, New York), 3 measures were taken with the first measure being discarded to account for any initial patient discomfort (as per laboratory protocol) and the remaining two readings were averaged. After a single blood draw, samples were assayed for liver function, lipid profiles, insulin and glucose by automated Clinical Chemistry Analyser (Unicel®DxC 800, Beckman Coulter, Inc., CA). Leptin, resistin, adiponectin, plasminogen activator inhibitor-1 (PAI-1) and soluble ICAM-1 were measured in duplicate using an in-house multiplex immunoassay 16 [23]; TNF-alpha and IL6 were measured using a high sensitivity Milliplex Kit (Millipore) and high sensitivity CRP was measured by immunonephelometry using Siemens Cardiophase hsCRP reagent and measured on a Siemens BNII instrument. Resting energy expenditure (REE) was measured using a ventilated hood calorimeter (Deltratrac II Metabolic Monitor, Datex-Engstrom Division, Helsinki, Finland), which was calibrated with a reference gas mixture of 95.00% O_2_ and 5.00% CO_2_. Oxygen consumption and carbon dioxide production was measured at one minute intervals for 30 minutes and averaged over the whole measurement period. The subjects were required to rest in a supine position for 20 minutes prior to the commencement of the test. The first five to ten minutes were excluded from the analysis to account for environmental adjustment by the subjects and gas adaptation in the hood. REE was calculated from the measured oxygen consumption and carbon dioxide production according to the formula by Weir [24]. A 4-day self-reported activity diary based on one described by Bouchard et al. (1983) was used to derive physical activity level (PAL), which divided a 24 hour day into 96 x 15 minute intervals and subjects were asked to record their activities during each time period [25]. On completion, each activity was categorised into nine levels according to their average energy costs, representing multiples of their respective metabolic equivalents (METs) and total daily METs values were calculated and averaged to give an estimated PAL [26]. Thus an individual’s total energy expenditure was estimated using the formula: EE = REE x PAL [27]. Lean cell mass was assessed using total body potassium (TBK) and this was converted to z-scores to adjust for age and height [28]. Bioelectrical impedance analysis (Bodystat 1500MDD) was used to estimate body fat. All outcomes measured at randomization (week 0) and at the end of the study period (week 12).

Demographic variables were recorded including ethnicity via a questionnaire administered to the parent, which offered the following categories; Caucasian, Asian, South Sea Islander, Aboriginal, Torres Strait Islander, Chinese or any other ethnic group. Social advantage was determined using the Australian Bureau of Statistics Socio-Economic Index for Areas measure of disadvantage. Each area represented by a postcode is ranked from most disadvantaged to least disadvantaged in terms of the economic and social wellbeing of the residents in that area [29]. We report social advantage after categorisation into tertiles.

### Interventions

The intervention comprised of an intensive treatment phase with the study dieticians providing five face-to-face counselling sessions for the subject and their carer (week 0,2,4,8 and 12) and two via telephone (weeks 6 and 10). Standardized manuals ensured the sessions followed a pre-determined path and used a client-centred approach with goal setting, problem solving and self-monitoring techniques. Each subject was given an individualized energy prescription based on a 20% energy reduction when compared to their estimated energy expenditure. A full description of the intervention delivered has been reported [19] and relied on a similar structured meal approach with a plate portion system (TEMPlate™) guiding portion size at main meals and an exchange system for other eating occasions. The plate templates had the appropriate size sections for the “structured low fat” (SLF, 55% carbohydrate, 20% protein, 25% fat) and the “structured modified carbohydrate” (SMC, 35% carbohydrate; 30% protein; 35% fat) to enable the desired macronutrient composition of the meal to be achieved. Controls were not provided with any dietary advice; however, they were offered the dietary program of their choice at the end of the study.

All groups received the Australian National Health and Medical Research Councils ‘Get out and get active’ booklet [30]. They were encouraged to set a goal to decrease sedentary behaviour. No formal exercise or activity was prescribed. Retention strategies included flexible appointment times, day-before text message appointment reminders and covering the cost of parking at the hospital.

The primary outcome variable was reduction of BMI z-score after 12 weeks of dietary intervention. Due to ethical considerations of withholding a potential treatment to a vulnerable control group, an unbalanced ratio of intervention and control was adopted with the allocation schedule weighted so that participants would be enrolled in an active diet group 82% of the time. Participants were allocated to treatment group using weighted randomisation, so that they were allocated to the group that minimized the gender/pubertal stage imbalance between groups with a probability of 0.8. If participants were not allocated to the group that minimized the imbalance, they were randomly assigned one of the two remaining treatment groups with equal probability. Allocation was conducted by the study statistician (RSW). Participants were informed of treatment group at their baseline appointment. Families, who were allocated to an active group, saw the study dietician (KAB) immediately. Due to the nature of the intervention, participant blinding to group allocation was not feasible.

### Sample Size

Sample size calculations were informed by a pilot study conducted by the same research team [17]. In the pilot study two diet intervention groups (low fat and modified carbohydrate) were followed for 12 weeks. At study completion the mean (standard deviation; SD) changes in BMI z-score were -0.20 (0.17) for the low fat group and -0.11 (0.08) for the modified carbohydrate group. In order to observe a clinically important difference in BMI z-score change between the two intervention groups of 0.09, with α = 0.05 and power of 80%, and assuming standard deviation of 0.12 (the overall SD from the pilot study), we required 29 individuals in each intervention group to complete the study. We calculated that if 12 week data was available on 12 participants from the control group, the study would have 80% power to detect a difference of change in BMI z-score of 0.13 between the control group and either of the diet intervention groups (α = 0.05). The difference of 0.13 was selected based on data extracted from a Cochrane Review of lifestyle interventions in obese adolescents and represents a clinically important change in BMI Z-score in this population. Based on these calculations we required 12 week data from 70 participants (29 in each of the low fat and modified carbohydrate groups and 12 in the control group). We assumed we would not be able to collect 12 week data from 20% of participants who began the study due to drop out (in pilot study 17% of participants dropped-out), and consequently intended to enrol 85 participants (35 in each of the low fat and modified carbohydrate groups, and 15 in the control group).

### Statistical methods

All analyses utilized the intention-to-treat approach, with participants analyzed in the group they were allocated to regardless of treatment compliance. Outcome variables are summarized using mean (standard deviation; SD) for continuous variables and n (%) for categorical variables. The distribution of variables was investigated, and where appropriate variables were transformed using the natural logarithm. Baseline characteristics were compared between the three diet groups by either a one-way analysis of variance with diet group as the single factor (continuous data) or Fisher’s Exact Test (categorical data). To compare the association between diet group and outcomes at the end of the intervention, we used an analysis of variance model with two factors: diet group and the outcome variable value at baseline. We included the value of the outcome variable at baseline to adjust for possible imbalances between diet groups. We tested the sensitivity of our results to missing data by using multiple imputation for the primary outcome. BMI z-score values were imputed conditional on the explanatory variables (age, gender, diet group, pubertal stage, and social position) using the Gaussian normal regression imputation method. Twenty data sets were imputed. These analyses were pre-specified. To further investigate our findings we conducted post-hoc analyses which considered the change from baseline to end of intervention within each of the three diet groups. We tested within-group change using Student’s t-distribution. Results of analyses are presented as mean difference (MD) and 95% confidence intervals (95%CI). A p-value of 0.017 was considered to indicate statistical significance (accounting for Bonferroni correction of the three pair-wise diet group comparisons). All analyses were conducted using Stata statistical software v. 11.1 (StataCorp, College Station, TX, USA).

## Results

**Fig 1:**
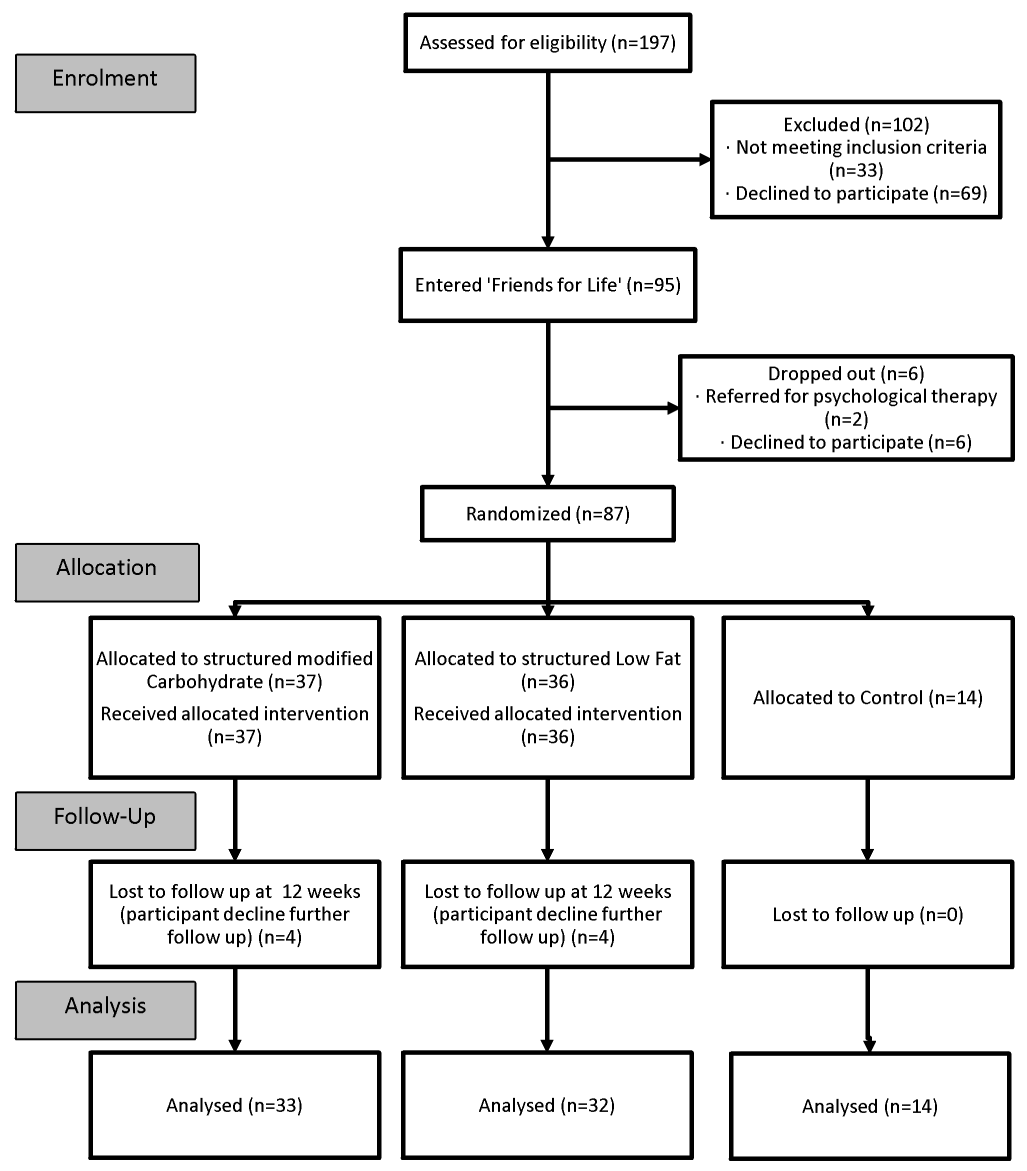
Recruitment and follow-up of study participants.

Participants were recruited continuously from February 2008 to October 2012, with follow-up completed by May 2013. A CONSORT flow chart of the study is shown in Fig 1. Of the 87 randomized, 79 (91%) children were successfully followed-up at 12 weeks.

Participants followed-up were similar to those who dropped-out in terms of age and gender (for those followed-up, 28% were male and mean (SD) age was 13.2 (2.0) years; for drop-outs, 25% were male and mean (SD) age was 13.6 (2.0) years; p = 1.00 for gender; p = 0.64 for age). Demographic, social and biochemical characteristics at baseline are presented in Table 1. Of particular note is the low body cell mass at baseline of participants as measured by TBK suggestive of sarcopenia visually masked by body fat.

**Table 1:** Demographic, social and biochemical characteristics of participants at baseline (n = 87).^a^^,^^b^

	Control	SLF	SMC	P-value
	(n = 14)	(n = 36)	(n = 37)	
Age, years	13.6 (1.9)	13.2 (2.1)	13.2 (1.9)	0.83
Female Gender	10 (71%)	26 (72%)	27 (73%)	1.00
Tanner stage				
I	1 (7%)	5 (14%)	4 (11%)	0.96
II	4 (29%)	7 (19%)	8 (22%)	
III	1 (7%)	6 (17%)	8 (22%)	
IV	4 (29%)	7 (19%)	8 (22%)	
V	4 (29%)	11 (31%)	9 (24%)	
Ethnicity				
Caucasian	10 (71%)	34 (94%)	33 (89%)	0.07
SE Asian	0 (0%)	1 (3%)	1 (3%)	
Aboriginal & Torres strait Islander/Pacific Islander	4 (29%)	1 (3%)	3 (8%)	
Social Advantage				
Lower-tertile	2 (14%)	7 (19%)	9 (24%)	0.57
Middle tertile	5 (36%)	14 (39%)	8 (22%)	
Upper tertile	7 (50%)	15 (42%)	20 (54%)	
BMI z-score	2.27 (0.43)	2.19 (0.39)	2.20(0.37)	0.78
Weight z-score	2.50 (0.76)	2.41(0.66)	2.43(0.53)	0.88
Height z-score	0.85 (0.87)	0.95 (1.07)	1.01 (1.02)	0.89
BCM/Height z-score	-0.47 (0.85)	-0.69 (0.92)	-0.31 (1.21)	0.30
% body fat (using BIA)	40.36 (5.31)	39.67 (6.38)	38.61 (4.55)	0.54
BMI (kg/m^2^)	35.17 (8.54)	32.62 (5.9)	32.47 (4.90)	0.33
Weight (kg)	94.42 (30.94)	86.63 (22.60)	86.23 (18.18)	0.48
Height (cm)	162.46 (8.83)	161.93 (11.21)	162.38 (11.34)	0.98
Waist circumference (cm)	112.44 (19.27)	105.30 (13.53)	104.99 (11.84)	0.20
Waist:Height ratio	0.69 (0.10)	0.65 (0.07)	0.65 (0.07)	0.18
Hypertension present	4 (29%)	5 (15%)	9 (24%)	0.46
HOMA-IR	2.5 (2.1)	1.7 (1.0)	1.8 (0.9)	0.08
Cholesterol				
Total (mmol/L)	4.5 (1.0)	4.4 (0.7)	4.4 (1.0)	0.90
HDL (mmol/L)	1.0 (0.3)	1.1 (0.2)	1.0 (0.2)	0.61
LDL (mmol/L)	2.9 (0.9)	2.8 (0.7)	2.7 (0.8)	0.84
VLDL (mmol/L)	0.6 (0.2)	0.5 (0.2)	0.6 (0.4)	0.47
Triglycerides (mmol/L)	1.3 (0.5)	1.2 (0.5)	1.4 (1.0)	0.53
Liver function				
ALT (U/L)	34.5 (31.3)	30.4 (22.5)	28.3 (21.6)	0.72
Adipokines/cytokines^c^				
Leptin (ng/mL)	3.9 (0.6)	3.9 (0.6)	3.8 (0.5)	0.77
Resistin (ng/mL)	2.3 (0.5)	2.2 (0.4)	2.1 (0.4)	0.51
Adiponectin (ng/mL)	2.0 (0.3)	2.2 (0.4)	2.1 (0.4)	0.19
Interleukin-6 (pg/mL)	2.0 (0.4)	1.9 (0.5)	1.9 (0.7)	0.82
TNF-alpha (pg/mL)	2.3 (0.7)	2.5 (0.6)	2.3 (0.9)	0.51
CRP (mg/L)	1.9 (0.7)	1.1 (1.1)	0.9 (0.9)	0.01
PAI1 (ng/mL)	2.8 (1.1)	2.8 (0.7)	2.8 (0.9)	0.98
ICAM (ng/mL)	5.6 (0.1)	5.5 (0.2)	5.6 (0.3)	0.71
Diet composition (% of energy)				
Protein	18 (2.4)	17 (3.2)	17 (2.7)	0.90
Fat	35 (3.3)	37 (5.2)	36 (5.7)	0.43
Carbohydrate	47 (4.5)	46 (6.2)	47 (6.6)	0.62
Resting energy expenditure (kcal/day)	1958 (321)	1914 (383)	1889 (299)	0.81
Physical Activity Level (PAL)	1.3 (0.1)	1.4 (0.2)	1.4 (0.1)	0.11

ALT = Alanine transaminase; BCM = body cell mass from total body potassium; BIA = bioelectrical impedance analysis technique; CRP = C-reactive protein; HDL = High-density lipoprotein; LDL = low-density lipoprotein; HOMA-IR = homeostasis model assessment-estimated insulin resistance; ICAM = Intercellular Adhesion Molecule; PAI-1 = plasminogen activator inhibitor-1; SLF = Structured Low Fat diet; SMC = Structured Modified Carbohydrate diet; VLDL = very low-density lipoprotein

^a^ Continuous variables presented as mean (standard deviation)

^b^ Categorical variables presented as frequency (percentage).

^c^ Adipokines/cytokines were transformed using the natural logarithm before analysis.

After 12 weeks of energy restriction, the mean (SD) BMI z-scores of the control, SLF and SMC groups were 2.29 (0.42), 2.10 (0.46) and 2.05 (0.41) respectively (Table 2). After adjusting for baseline BMI z-scores, there was no significant difference at the end of intervention between BMI z-scores in the SLF and SMC groups, MD (95% CI) = 0.00 (-0.05, 0.04) p = 0.83; but there was a significant difference between both the SLF and control (-0.13 (-0.18, -0.07) p<0.001) and SMC and control (-0.14 (-0.19, -0.09) p<0.001) diet groups. When a sensitivity analysis was undertaken on the primary outcome, BMI z-score, the effect estimates for BMI z-score did not change substantively for any of the three pairwise comparisons: SLF vs. SMC, MD (95% CI) = -0.01 (-0.06, 0.03), p = 0.57; SLF vs. control, -0.12 (-0.18, -0.06), p<0.001; SMC vs. control, -0.14 (-0.20, -0.08), p<0.001. Similar patterns of results were observed for the outcomes weight z-score, BMI, weight, waist circumference and waist:height ratio, where a non-significant difference was observed between the SLF and SMC groups, but a statistically significant difference existed between the control diet group and each of the intervention diet groups. Change from baseline to end of intervention within diet groups for anthropometric and body composition measures are displayed in S2 Table. For the outcomes BMI z-score and weight z-score, we observed statistically significant decreases in the SLF and SMC groups (all P<0.001), whereas we observed a slight increase in the control group. Similar findings hold for BMI, weight, waist circumference and waist:height ratio.

**Table 2:** Anthropometric, body and diet composition at end of intervention (n = 79). Between-diet group differences calculated using linear regression with adjustment of value of outcome at baseline.^a^^,^^b^

	Control	SLF	SMC		Control vs. SLF	Control vs. SMC	SLF vs. SMC
	(n = 14)	(n = 32)	(n = 33)				
	Mean (SD)	Mean (SD)	Mean (SD)	P-value	MD (95%CI); P	MD (95%CI); P	MD (95%CI); P
BMI z-score	2.29 (0.42)	2.10 (0.46)	2.05 (0.41)	<0.001	-0.13 (-0.18, -0.07); <0.001	-0.14 (-0.19, -0.09); <0.001	-0.00 (-0.05, 0.04);0.83
Weight z-score	2.52 (0.75)	2.32 (0.70)	2.25 (0.55)	<0.001	-0.15 (-0.21, -0.10); <0.001	-0.17 (-0.24, -0.09); <0.001	-0.01 (-0.06, 0.05);0.83
Height z-score	0.88 (0.90)	1.04 (1.08)	1.00 (1.09)	0.22	-0.05 (-0.11, 0.02);	-0.01 (-0.08, 0.06);	0.04 (-0.01, 0.10);
					0.14	0.83	0.15
% body fat (BIA)	42.98 (4.26)	39.54 (5.22)	38.21 (5.26)	0.01	-2.99 (-5.50, -0.48);	-3.89 (-6.72, -1.06);	-0.77 (-2.65, 1.11);
					0.02	0.008	0.42
BCM/ Height z-score	-0.86 (0.80)	-0.79 (0.93)	-0.54 (1.15)	0.58	0.20 (-0.28, 0.69);	0.19 (-0.23, 0.61);	-0.05 (-0.38, 0.28);
					0.41	0.37	0.77
BMI (kg/m^2^)	35.74 (8.66)	31.88 (6.17)	30.82 (4.85)	<0.001	-1.58 (-2.06, -1.11);	-1.75 (-2.43, -1.07);	-0.15 (-0.66, 0.35);
					<0.001	<0.001	0.54
Weight (kg)	96.88 (31.00)	86.26 (23.38)	83.03 (17.73)	<0.001	-4.15 (-5.50, -2.81);	-4.51 (-6.41, -2.60);	-0.22 (-1.62, 1.19);
					<0.001	<0.001	0.76
Height (cm)	163.50 (8.45)	163.46 (11.40)	163.55 (11.36)	0.44	-0.03 (-0.56, 0.49);	0.25 (-0.38, 0.89);	0.28 (-0.21, 0.78);
					0.90	0.43	0.25
Waist circumference (cm)	113.26 (19.54)	102.88 (14.96)	101.48 (12.03)	0.005	-3.07 (-4.54, -1.61);	-3.74 (-6.29, -1.19);	-0.19 (-1.98, 1.60);
					<0.001	0.005	0.83
Waist:Height ratio	0.69 (0.10)	0.63 (0.08)	0.62 (0.07)	0.005	-0.02 (-0.03, -0.01);	-0.02 (-0.04, -0.01);	-0.00 (-0.01, 0.01);
					<0.001	0.006	0.75
Protein (%E)	20.0 (3.0)	21.5 (4.5)	24.1 (5.3)	0.04	1.6 (-2.1, 5.4); 0.38	5.1 (0.5, 9.7); 0.03	3.6 (0.5, 6.7); 0.02
Fat (%E)	37.1 (4.7)	31.9 (5.9)	36.9 (6.7)	0.14	-5.7 (-11.3, 0.0); 0.05	-1.4 (-6.9, 4.1); 0.60	4.3 (0.3, 8.3); 0.04
Carbohydrate (%E)	42.9 (3.0)	46.6 (5.7)	39.0 (7.6)	0.04	4.1 (-0.8, 9.0); 0.10	-3.3 (-9.4, 2.7); 0.27	-7.7 (-11.9, -3.6); 0.01

BCM = body cell mass from total body potassium; BIA = bioelectrical impedance analysis; E = energy; SLF = Structured low fat diet; SMC = Structured modified carbohydrate diet

^a^ Between-diet group differences presented as mean difference (MD); (95% Confidence Interval); P-value.

^b^ Number of paired measurements analyzed for control group, n = 14, except BCM/ Height z-score (n = 13), diet composition outcomes (n = 6); for SLF group, n = 32 except except BCM/ Height z-score (n = 29), diet composition outcomes (n = 19); for SMC group n = 33, except diet composition outcomes (n = 17).

There were a number of differences in biochemical parameters evident between the control and intervention groups (Table 3). Insulin resistance (HOMA-IR) was reduced in both intervention groups and was statistically different from the control group. Both SLF and SMC groups emerged with significantly lower leptin levels than the control group. Change from baseline to end of intervention within diet groups for biochemistry and energy expenditure measures are displayed in S3 Table.

**Table 3:** Biochemistry and energy expenditure at end of intervention (n = 79). Between-diet group differences calculated using linear regression with adjustment for value of outcome at baseline. ^a^^,^^b^

	Control	SLF	SMC		Control vs. SLF	Control vs. SMC	SLF vs. SMC
	(n = 14)	(n = 32)	(n = 33)				
	Mean (SD)	Mean (SD)	Mean (SD)	P-value	MD (95%CI); P	MD (95%CI); P	MD (95%CI); P
HOMA IR	2.7 (1.1)	1.5 (0.9)	1.6 (0.9)	0.03	-0.7 (-1.2, -0.2); 0.009	-0.7 (-1.3, -0.2); 0.01	0.0 (-0.3, 0.4); 0.79
Cholesterol							
Total (mmol/L)	4.4 (1.1)	4.2 (0.7)	4.2 (0.8)	0.20	-0.2 (-0.5, 0.0); 0.06	-0.2 (-0.5, 0.1); 0.30	0.1 (-0.1, 0.2); 0.40
HDL (mmol/L)	1.1 (0.4)	1.0 (0.2)	1.0 (0.2)	0.20	-0.1 (-0.2, 0.0); 0.16	0.0 (-0.1, 0.1); 0.71	0.1 (0.0, 0.1); 0.15
LDL (mmol/L)	2.8 (0.9)	2.6 (0.6)	2.6 (0.7)	0.58	-0.1 (-0.3, 0.1); 0.28	-0.1 (-0.4, 0.2); 0.49	0.0 (-0.1, 0.2); 0.74
VLDL (mmol/L)	0.5 (0.2)	0.5 (0.2)	0.5 (0.3)	0.46	0.1 (0.0, 0.1); 0.27	0.0 (-0.1, 0.1); 0.94	0.0 (-0.1, 0.0); 0.27
Triglycerides (mmol/L)	1.2 (0.7)	1.2 (0.4)	1.2 (0.5)	0.84	0.1 (-0.1, 0.3); 0.60	0.0 (-0.3, 0.3); 0.98	0.0 (-0.2, 0.1); 0.58
Liver function							
ALT (U/L)	26.0 (8.1)	27.0 (14.5)	23.9 (15.5)	0.75	1.8 (-6.9, 10.4); 0.69	-0.2 (-8.7, 8.3); 0.96	-2.0 (-7.9, 3.9); 0.50
Adipokines/cytokines^c^							
Leptin (ng/mL)	4.1 (0.5)	3.6 (0.7)	3.5 (0.7)	0.004	-0.4 (-0.7, -0.2); 0.001	-0.4 (-0.7, -0.1); 0.005	0.0 (-0.2, 0.2); 0.75
Resistin (ng/mL)	2.6 (1.0)	2.2 (0.6)	2.2 (0.5)	0.49	-0.2 (-0.7, 0.2); 0.35	-0.2 (-0.6, 0.2); 0.36	0.0 (-0.2, 0.2); 0.99
Adiponectin (ng/mL)	2.0 (0.2)	2.2 (0.4)	2.2 (0.4)	0.09	0.1 (-0.1, 0.2); 0.33	0.1 (0.0, 0.3); 0.04	0.1 (0.0, 0.2); 0.13
Interleukin-6 (pg/mL)	1.8 (0.5)	1.7 (0.6)	1.7 (0.8)	0.76	0.0 (-0.3, 0.3); 0.97	-0.1 (-0.4, 0.3); 0.76	-0.1 (-0.4, 0.2); 0.47
TNF-alpha (pg/mL)	2.5 (1.1)	2.3 (0.6)	2.3 (0.8)	0.36	-0.3 (-0.8, 0.2); 0.22	-0.2 (-0.7, 0.3); 0.43	0.1 (-0.1, 0.3); 0.37
CRP (mg/L)	1.6 (1.2)	0.9 (0.9)	0.8 (1.0)	0.55	-0.3 (-1.0, 0.2); 0.24	-0.3 (-1.1, 0.5); 0.43	0.1 (-0.3, 0.4); 0.72
PAI-1 (ng/mL)	3.0 (0.9)	2.8 (0.8)	2.8 (0.8)	0.93	0.0 (-0.4, 0.4); 0.99	-0.1 (-0.5, 0.3); 0.70	0.0 (-0.3, 0.2); 0.74
ICAM (ng/mL)	5.3 (0.8)	5.4 (0.2)	5.6 (0.2)	0.09	0.2 (-0.1, 0.5); 0.19	(-0.1, 0.6); 0.10	0.1 (0.0, 0.2); 0.01
Resting energy expenditure (kcal/d)	2011 (361)	1852 (403)	1893 (333)	0.17	-132 (-237, -28); 0.01	-12 (-129, 106); 0.84	116 (25, 208); 0.01
Physical activity level	1.4 (0.2)	1.4 (0.1)	1.5 (0.1)	0.25	0.0 (-0.1, 0.1); 0.78	0.0 (-0.1, 0.1); 0.90	0.0 (-0.1, 0.1); 0.67

ALT = Alanine transaminase; BCM = body cell mass from total body potassium; BIA = bioelectrical impedance analysis technique; CRP = C-reactive protein; HDL = High-density lipoprotein; LDL = low-density lipoprotein; HOMA-IR = homeostasis model assessment-estimated insulin resistance; ICAM = Intercellular Adhesion Molecule; PAI-1 = plasminogen activator inhibitor-1; SLF = Structured Low Fat diet; SMC = Structured Modified Carbohydrate diet; VLDL = very low-density lipoprotein

^a^ Between-diet group differences presented as mean difference (MD); (95% Confidence Interval); P-value.

^b^ Number of paired measurements analyzed for control group, n = 14, except HOMA IR (n = 12), all cholesterol outcomes (n = 12), triglycerides (n = 12), liver function (n = 11), adipokines/cytokines except ICAM (n = 12), ICAM (n = 9), physical activity level (n = 6); for SLF group, n = 32 except ICAM (n = 23), physical activity level (n = 19); for SMC group n = 33, except HOMA IR (n = 32), all cholesterol outcomes (n = 32), triglycerides (n = 32), liver function (n = 31), adipokines/cytokines except ICAM (n = 32), ICAM (n = 24), physical activity level (n = 20)

^c^Adipokines/cytokines were transformed using the natural logarithm before analysis.

Diet changes are described in Table 2. At randomization, there were no significant differences in macronutrient content of the diets between groups (mean %energy: carbohydrate 47%, protein 22%, fat 34%) and after 12 weeks the SLF group had significantly reduced their fat intake by 4%, the SMC group had a significant decrease in carbohydrate by 9% and significant increase in protein by 7%. There were no statistically significant differences pre- and post-intervention in activity measures (overall PAL, hours of screen time and hours of sedentary behaviour) between or within groups.

At baseline 16/87 (18%) participants met the criteria International Diabetes Federation definition for the Metabolic Syndrome^21^, this had reduced to 8/79 (10%) completers (with no significant group differences). Hypertension remained present in 1 (7%) control, 6 (19%) SLF and 5 (15%) in the SMC group.

No physical adverse events were reported. No significant change in psychological parameters was demonstrated via the ADIS interviews.

## Discussion

Our findings demonstrate that a both the SLF and a SMC diet have the same short term benefits in terms of weight loss, body composition change and lipid profile, and both are better than not intervening. This challenges the current practice of only recommending a low fat/higher carbohydrate diet for treatment of childhood obesity. The intensive nature of the ‘Eat Smart’ protocol was designed to have the maximum impact in a short time frame. Thus these results can be viewed in terms of what can be optimally expected of a dietary intervention using normal food and are comparable with a 2012 meta-analysis by Ho et al. [31] which showed the mean effect of 33 lifestyle interventions was a BMI z-score reduction of -0.10 (95%CI -0.18 to -0.02).

Given the severity of the obesity in these young people, we wished to ascertain whether a moderate reduction in carbohydrate with a higher protein intake could be as effective as a low fat eating pattern. Our results concur with the report by Demol. et al. [32] that suggest that both SMC and SLF diets result in similar reductions in biochemical indices of cardiovascular risk, at least in the short term. These data suggest that the rigid adoption of a low fat eating regime is not necessarily the only dietary pattern that could result in longer term cardiovascular protection. Flexibility in terms of finding a reduced energy intake eating pattern that is the most acceptable to the individual is appropriate. However, the rapid weight loss seen by using very low carbohydrate or protein modified fasts was not experienced by our participants and as such it provides evidence that a more severe carbohydrate restriction, at least in the initial phases of a weight loss attempt, would be required. We demonstrate that a clinically relevant reduction in adiposity can realistically be achieved in a group of obese youth presenting for weight management at a specialist public children’s hospital. The upward weight trajectory of the untreated control group strengthens the case that doing nothing is not an option.

This study contributes to the understanding of body composition alterations in adolescents following energy restricted diets. Here we demonstrate a significant loss of body fat (3–4 percentage points) with concurrent preservation of lean tissue, the optimal outcome in body composition change during weight loss. Using the measurement of total body potassium, an accurate measure of body cell mass independent of body water, we describe the relatively low lean mass of all the participants at baseline. In adults, sarcopenia in the presence of obesity is documented and its relationship with functional impairment and increased cardiovascular risk is emerging [33, 34]. Consequently, the addition of resistance strength training to preserve lean tissue further may well be of benefit and perhaps acceptable to teenagers, as it is in adults. This highlights the critical need for any intervention to preserve lean mass whilst targeting a reduction in body fat but importantly that a moderate increase in protein as demonstrated here was insufficient to completely preserve lean mass. Weight management programs aimed at children should include body composition measurement to monitor the effect on body compartment changes which are independent of shifts in body water and not rely solely on the relatively anthropometric instruments [35].

The use of structure such as portion size control and managed eating episodes are crucial elements of success in weight control. ‘Eat Smart’ was designed specifically for this difficult target group and their parents who wished to have a system that would enable them to plan together for meals and snacks with some flexibility but with clear limits. This technique also assists the health care professional in monitoring adherence to goals [36]. The reduction in the indicators of the metabolic syndrome at the end of the study period confirms that improvement in weight status can produce meaningful reduction in cardiovascular risk factors, but clearly some require more intensive intervention or further weight loss to reduce their blood pressure to acceptable levels. There was no evidence that the relative increase in dietary fat experienced as a result of reducing carbohydrates had any adverse effect on inflammatory markers which may be mediated by weight and body fat changes. Loss of body fat is the likely facilitator of the significant drop in leptin in the active groups compared to control. Overall, the metabolic impact of such dietary modifications was not profound although the study was not powered to detect small differences in these parameters.

Treatment of childhood obesity has been described as complex and fraught with difficulties [37]. ‘Eat Smart’ used a preparatory psychological group program, FRIENDS for life^TM^, to ensure that all of those randomized did not meet clinical criteria for anxiety, depression and disordered eating, common problems that we and others have identified in the obese child although not often addressed prior to intervention [17, 38]. All randomized participants received advice and support in behaviour change processes prior to beginning their weight management journey. This unique step may have contributed to our low attrition rate (Fig 1). The higher proportion of predominately Caucasian girls (69%), living in lower socio-demographic areas situates this study group for the purposes of generalizability. A paper which is a reflective analysis of completers across the ‘Eat Smart’ studies characterised what factors were predictive of successful weight loss and demonstrated that those who were most successful were those enjoying higher social advantage and presenting with a lower BMI [39].

## Strengths and Limitations

The evaluation of these two structured diets has a number of strengths that include the use of a control group and the successful completion of psychological preparedness program, which ensured that all randomized participants were similarly ready to adopt lifestyle change. The dietary patterns tested were piloted for feasibility and acceptability in young people which resulted in two different macronutrient profiles and therefore all 3 macronutrients being different in both intervention groups. The protocol of intensive follow up and dietician debriefing sessions along with a structured portion control system was designed to maintain consistency of the dietary components. The imprecision of dietary measures and accuracy of reporting, issues for most diet related interventions, must be acknowledged as a limitation. We report energy-adjusted nutrients to reduce measurement error as it removes extraneous variation that results from populations, such as adolescents, where differences in individual energy requirements, due to their different body sizes and physical activity levels are inevitable [40]. Also the short term intervention period must be regarded as a limitation. Obviously longer term follow up is needed to establish if food choices are maintained over time, however, we accept that after 12 weeks, as in adults [40, 41], weight loss has most likely peaked and longer duration of intensive lifestyle only advice may offer little additive benefit for weight change [4].

## Conclusion

The burgeoning issue of adolescent obesity requires availability of effective treatment options that are acceptable to both the adolescent and their caregivers. This study demonstrates that macronutrients may not be as critical as methodologies that control overall energy intake, thus supporting the impact that more individualization and tailoring of dietary prescription for young people seeking weight management can be recommended which should increase compliance in the longer term.

## Supporting Information

S1 Table CONSORT 2010 checklist. (DOC)

S2 Table Changes in anthropometric and body composition measures from baseline to end of intervention within diet groups. (DOCX)

S3 Table Changes in biochemistry and energy expenditure from baseline to end of intervention within diet groups. (DOCX)

S1 Text The Eat Smart Study trial protocol. (PDF)
