# Supplementary material for: A Randomized Controlled Trial of Two Different Macronutrient Profiles on Weight, Body Composition and Metabolic Parameters in Obese Adolescents Seeking Weight Loss
Source: PLoS One. 2016 Mar 29;11(3):e0151787. doi: 10.1371/journal.pone.0151787 (PMC4811557; doi:10.1371/journal.pone.0151787)
Supplement: S2 Table — (DOCX) [file pone.0151787.s002.docx]

**S2 Table. Changes in anthropometric and body composition measures from baseline to end of intervention within diet groups (n=79).**

|  | **Control (n=14)** | | | **SLF (n=32)** | | | **SMC (n=33)** | | |
| --- | --- | --- | --- | --- | --- | --- | --- | --- | --- |
|  | **Baseline** | **12 weeks** | **Change** | **Baseline** | **12 weeks** | **Change** | **Baseline** | **12 weeks** | **Change** |
|  | **mean(SD)** | **mean(SD)** | **MD(95%CI);P** | **mean(SD)** | **mean(SD)** | **MD(95%CI);P** | **mean(SD)** | **mean(SD)** | **MD(95%CI);P** |
| BMI Z-score | 2.27 (0.43) ^†^ | 2.29 (0.42) | 0.02 (0.00, 0.03); 0.06 | 2.21 (0.38) | 2.10 (0.46) | -0.12 (-0.15, -0.08); <0.001 | 2.18 (0.38) | 2.05 (0.41) | -0.13 (-0.16, -0.09); <0.001 |
| Weight Z-score | 2.50 (0.76) | 2.52 (0.75) | 0.02 (0.00, 0.05); 0.06 | 2.46 (0.65) | 2.33 (0.70) | -0.13 (-0.17, -0.10); <0.001 | 2.39 (0.54) | 2.25 (0.55) | -0.14 (-0.19, -0.10); <0.001 |
| Height Z-score | 0.85 (0.87) | 0.88 (0.90) | 0.03 (-0.02, 0.07); 0.22 | 1.06 (1.09) | 1.04 (1.08) | -0.02 (-0.05, 0.02); 0.30 | 0.97 (1.07) | 1.00 (1.09) | 0.02 (-0.02, 0.06); 0.32 |
| %body fat (BIA) | 40.36 (5.31) | 42.98 (4.26) | 2.62 (-0.91, 6.15); 0.13 | 39.38 (6.64) | 39.48 (5.30) | -0.10 (-1.43, 1.64); 0.89 | 38.28 (4.45) | 37.98 (5.18) | -0.29 (-1.77, 1.19)l 0.69 |
| BCM/Height Z score | -0.47 (0.85) | -0.86 (0.80) | -0.39 (-0.90, 0.12); 0.12 | -0.68 (0.88) | -0.79 (0.93) | -0.11 (-0.39, 0.17); 0.44 | -0.30 (1.24) | -0.54 (1.15) | -0.24 (-0.46, -0.02); 0.03 |
| BMI (kg/m^2^) | 35.17 (8.54) | 35.74 (8.66) | 0.57 (0.29, 0.86); 0.001 | 32.92 (6.07) | 31.88 (6.17) | -1.03 (-1.32, -0.75); <0.001 | 32.01 (4.75) | 30.82 (4.85) | -1.19 (-1.60, -0.78); <0.001 |
| Weight (kg) | 94.42 (30.94) | 96.88 (31.00) | 2.46 (1.56, 3.36); <0.001 | 87.97 (23.20) | 86.26 (23.38) | -1.71 (-2.51, -0.91); <0.001 | 84.92 (18.12) | 83.03 (17.73) | -1.89 (-3.06, -0.72); <0.001 |
| Height (cm) | 162.46 (8.83) | 163.50 (8.45) | 1.04 (0.63, 1.46); <0.001 | 162.45 (11.67) | 163.46 (11.40) | 1.01 (0.68, 1.34); <0.001 | 162.25 (11.56) | 163.54 (11.36) | 1.30 (0.90, 1.70); <0.001 |
| Waist circumference (cm) | 112.44 (19.27) | 113.26 (19.54) | 0.82 (-0.14, 1.78); 0.09 | 105.38 (14.02) | 102.88 (14.96) | -2.50 (-3.39, -1.61); <0.001 | 104.17 (12.02) | 101.48 (12.03) | -2.70 (-4.25, -1.14); 0.001 |
| Waist:Height ratio | 0.69 (0.10) | 0.69(0.10) | 0.00 (-0.01. 0.01); 0.75 | 0.65 (0.08) | 0.63(0.08) | -0.02 (-0.03, -0.01); <0.001 | 0.64 (0.07) | 0.62(0.07) | -0.02 (-0.03, -0.01); <0.001 |

BCM = body cell mass from total body potassium; BIA = bioelectrical impedance analysis technique; CI = confidence interval; MD = mean difference; SLF = Structured Low Fat diet; SMC = Structured Modified Carbohydrate diet

Number of paired measurements analyzed for control group, n=14, except BCM/Height Z score (n=13); for SLF group, n=32 except % body fat (n=31), BCM/Height Z score (n=29); for SMC group n = 33, except % body fat (n=32)
