# Supplementary material for: A Randomized Controlled Trial of Two Different Macronutrient Profiles on Weight, Body Composition and Metabolic Parameters in Obese Adolescents Seeking Weight Loss
Source: PLoS One. 2016 Mar 29;11(3):e0151787. doi: 10.1371/journal.pone.0151787 (PMC4811557; doi:10.1371/journal.pone.0151787)
Supplement: S3 Table — (DOCX) [file pone.0151787.s003.docx]

**S3 Table: Changes in biochemistry and energy expenditure from baseline to end of intervention within diet groups (n=79).**

|  | **Control (n=14)** | | | **SLF (n=32)** | | | **SMC (n=33)** | | |
| --- | --- | --- | --- | --- | --- | --- | --- | --- | --- |
|  | **Baseline** | **12 weeks** | **Change** | **Baseline** | **12 weeks** | **Change** | **Baseline** | **12 weeks** | **Change** |
|  | **mean(SD)** | **mean(SD)** | **MD(95%CI);P** | **mean(SD)** | **mean(SD)** | **MD(95%CI);P** | **mean(SD)** | **mean(SD)** | **MD(95%CI);P** |
| HOMA IR | 2.7 (2.2) | 2.7 (1.1) | -0.1 (-1.1, 1.0); 0.92 | 1.7 (1.0) | 1.5 (0.9) | -0.2 (-0.4, 0.0); 0.05 | 1.7 (0.9) | 1.6 (0.9) | -0.2 (-0.4, 0.1); 0.28 |
| Cholesterol |  |  |  |  |  |  |  |  |  |
| Total (mmol/L) | 4.4 (1.0) | 4.4 (1.1) | 0.0 (-0.3, 0.3); 0.87 | 4.3 (0.7) | 4.2 (0.7) | -0.2 (-0.3, -0.1); <.001 | 4.2 (0.9) | 4.2 (0.8) | -0.1 (-0.3, 0.1); 0.19 |
| HDL (mmol/L) | 1.1 (0.4) | 1.1 (0.4) | 0.0 (-0.1, 0.1); 0.53 | 1.1 (0.3) | 1.0 (0.2) | 0.0 (-0.1, 0.0); 0.14 | 1.0 (0.2) | 1.0 (0.2) | 0.0 (0.0, 0.1); 0.32 |
| LDL (mmol/L) | 2.8 (0.8) | 2.8 (0.9) | 0.0 (-0.3, 0.3); 0.95 | 2.7 (0.6) | 2.6 (0.6) | -0.1 (-0.2, 0.0), 0.01 | 2.7 (0.8) | 2.6 (0.7) | -0.1 (-0.2, 0.1); 0.23 |
| VLDL (mmol/L) | 0.6 (0.2) | 0.5 (0.2) | -0.1 (-0.2, 0.0); 0.25 | 0.5 (0.2) | 0.5 (0.2) | 0.0 (0.0, 0.1); 0.90 | 0.6 (0.4) | 0.5 (0.3) | -0.1 (-0.1, 0.0); 0.15 |
| Triglycerides (mmol/L) | 1.3 (0.5) | 1.2 (0.7) | -0.1 (-0.3, 0.2); 0.47 | 1.2 (0.5) | 1.2 (0.4) | 0.0 (-0.1, 0.1); 0.80 | 1.3 (0.8) | 1.2 (0.5) | -0.1 (-0.2, 0.1); 0.32 |
| Liver function |  |  |  |  |  |  |  |  |  |
| ALT (U/L) | 33.6 (33.9) | 25.8 (8.5) | -7.8 (-30.6, 15.0); 0.46 | 31.0 (23.1) | 27.0 (14.5) | -4.0 (-11.0, 3.0); 0.25 | 29.3 (22.6) | 24.3 (15.7) | -5.0 (-10.6, 0.6); 0.08 |
| Adipokines/cytokines^§^ |  |  |  |  |  |  |  |  |  |
| Leptin (ng/mL) | 4.0 (0.8) | 4.1 (0.5) | 0.1 (0.0, 0.3); 0.10 | 3.9 (0.7) | 3.6 (0.7) | -0.3 (-0.4, -0.2); <.001 | 3.8 (0.5) | 3.5 (0.7) | -0.3 (-0.4, -0.1); 0.001 |
| Resistin (ng/mL) | 2.4 (0.5 | 2.6 (1.0) | 0.2 (-0.3, 0.7); 0.45 | 3.9 (0.7) | 2.2 (0.6) | 0.0 (-0.2, 0.2); 0.75 | 2.1 (0.4) | 2.2 (0.5) | 0.0 (-0.1, 0.2); 0.56 |
| Adiponectin (ng/mL) | 2.0 (0.3) | 2.0 (0.2) | 0.0 (-0.1, 0.1); 0.74 | 2,2 (0.4) | 2.2 (0.4) | 0.1 (0.0, 0.1); 0.13 | 2.1 (0.4) | 2.2 (0.4) | 0.1 (0.1, 0.2); 0.001 |
| Interleukin-6 (pg/mL) | 2.0 (0.5) | 1.8 (0.5) | -0.2 (-0.5, 0.1); 0.16 | 1.9 (0.5) | 1.7 (0.6) | -0.1 (-0.3, 0.0); 0.15 | 1.9 (0.6) | 1.7 (0.8) | -0.2 (-0.4, 0.0); 0.03 |
| TNF-alpha (pg/mL) | 2.3 (0.5) | 2.5 (1.1) | 0.2 (-0.5, 0.9); 0.56 | 2.5 (0.6) | 2.3 (0.6) | -0.1 (-0.3, 0.0); 0.08 | 2.3 (0.8) | 2.3 (0.8) | 0.0 (-0.2, 0.2); 0.93 |
| CRP (mg/L) | 1.9 (0.8) | 1.6 (1.2) | -0.3 (-1.2, 0.6); 0.42 | - 1. (1.1) | 0.9 (0.9) | -0.3 (-0.6, 0.0); 0.03 | 1. (0.8) | 0.8 (1.0) | -0.2 (-0.5, 0.2); 0.30 |
| PAI-1 (ng/mL) | 3.0 (1.1) | 3.0 (0.9) | 0.0 (-0.5, 0.5); 0.99 | 2.7 (0.7) | 2.8 (0.8) | 0.0 (-0.1, 0.2); 0.59 | 2.8 (0.9) | 2.8 (0.8) | 0.0 (-0.2, 0.3); 0.98 |
| ICAM (ng/mL) | 5.6 (0.1) | - 1. (0.8) | -0.2 (-0.8, 0.3); 0.34 | 5.5 (0.2) | 5.4 (0.2) | 0.0 (-0.1, 0.0); 0.22 | 5.5 (0.3) | 5.6 (0.2) | 0.0 (0.0, 0.1); 0.27 |
| Resting energy expenditure  (kJ/kg/Day) | 1958 (321) | 2011 (361) | 52 (-25, 130); 0.17 | 1931 (389) | 1852 (403) | -79 (-140, -17); 0.01 | 1851 (281) | 1893 (333) | 42 (-27, 110); 0.23 |
| Physical Activity Level | 1.3 (0.1) | 1.4 (0.2) | 0.0 (-0.1, 0.2); 0.62 | 1.4 (0.2) | 1.4 (0.1) | 0.0 (0.0, 0.1); 0.44 | 1.5 (0.1) | 1.5 (0.1) | 0.0 (-0.1, 0.0); 0.55 |

^§^ Adipokines/cytokines were transformed using the natural logarithm before analysis.

ALT = Alanine transaminase; BCM = body cell mass from total body potassium; BIA = bioelectrical impedance analysis technique; CI = confidence interval; CRP = C-reactive protein; HDL = High-density lipoprotein; LDL = low-density lipoprotein; HOMA-IR = homeostasis model assessment-estimated insulin resistance; ICAM = Intercellular Adhesion Molecule; MD = mean difference; PAI-1 = plasminogen activator inhibitor-1; SLF = Structured Low Fat diet; SMC = Structured Modified Carbohydrate diet; VLDL = very low-density lipoprotein

Number of paired measurements analyzed for control group, n=14, except HOMA IR (n=12), all cholesterol outcomes (n=12), triglycerides (n=12), liver function (n=11), adipokines/cytokines except ICAM (n=12), ICAM (n=9), physical activity level (n=6); for SLF group, n=32 except ICAM (n=23), physical activity level (n=19); for SMC group n = 33, except HOMA IR (n=32), all cholesterol outcomes (n=32), triglycerides (n=32), liver function (n=31), adipokines/cytokines except ICAM (n=32), ICAM (n=24), physical activity level (n=20)
